# Mr. Dashwood's Case of Tetanus

**Published:** 1806-06

**Authors:** C. Dashwood

**Affiliations:** Surgeon. Beccles


### *To the Editors of the Medical and Phyſical Journal.*

GENTLEMEN,

I Beg leave to submit the following case of Tetanus to your perusal, it being one of those diseases which seldom occur in this country, except from external injury; and having in the present instance been successfully treated by mercury, opium, and cold affusion.

*Beccles,* I am, &c.

*April* 12, 1806. C. DASHWOOD, Surgeon.

---

The patient alluded to is a female in her twenty-sixth year, of dark complexion, and in every respect previous to this attack perfectly healthy. She dates her complaint from cold, caught by coming down into a damp shop early in the morning half dressed; as, soon after, she felt a pain and stiffness in her neck and under jaw. This continued gradually to increase; but supposing it only a common cold, she kept herself warm, and took some trifling remedies for three days.

On the 1st of March, I was sent for, and then found her feverish, and complaining of stiffness about the neck, shoulders, and inferior maxillary bone. The bowels being costive, I ordered a gr. viij calomel bolus and opening draught, with a blister to be applied on each side the jaw, and another between her shoulders.

The next morning, March 2, finding her bowels had not been moved, an infusion of senna and salts was ordered to be taken every two hours till it operated; and as the symptoms of tetanus were evidently increasing, I ordered her, as soon as possible, to be put into a warm bath for three quarters of an hour, and again in the evening. During the time she was in the bath, she was much easier, and, to use her own expression, wished she could be always in it, for she felt to ail nothing whilst there. Each time after she was put to bed, sixty drops of tinct. opii were given, and a grain of extr. of opium every four hours.

March 3. Visited her at eleven o'clock A. M.; she had a restless night, and five motions from the opening mixture; opium increased, and warm bath repeated twice.

March 4. Saw her at twelve; she had not had more than two hours sleep in the night. The warm bath and opium were repeated, but finding her spasms still increasing, ing, her mouth close shut, and her head forcibly and rigidly drawn back, I was desirous of further advice, and therefore requested Mr. Crowfoot of this place to visit her with me in the afternoon, to which he obligingly consented. We then found her no better; but, as she expressed so much ease from the warm bath, it was agreed to use it again, and repeat the opium with the addition of four grains of ammonia ppt. and two of calomel in each dose.

March 5. No sleep last night; and this morning, in making an attempt to open her mouth in my presence, the nurse put the end of a tea-spoon between her teeth, which produced such a violent spasm of the jaw that a piece of one of the incisor teeth snapped off. The warm bath was again repeated, and the opium continued with gr. iv. of calomel. On returning home, I accidentally met Mr. W. H. Crowfoot, nephew of the former gentleman, who enquired after my patient, and mentioned his having seen, during his studies at Guy's and St. Thomas's Hospitals, a case of tetanus successfully treated by cold affusion. After some further conversation upon the subject, I requested he would accompany me in my next visit; when we found her still worse, and therefore proposed to her and her friends the application of cold water: but their prejudices were so great against it, that recourse was once more had to the warm bath, with the medicines as before, repeated every three hours; at the same time we assured them, that if she was not better in the morning, the application of cold water would be the only means of relieving her.

March 6. This morning at ten, I visited her in company with Mr. W. H. C.; she had had two motions in the night, but less power over the jaw, with still greater rigidity of the back. With some difficulty we persuaded her friends to consent to the cold affusion, which we agreed to apply at twelve o'clock, when the two Mr. C's accompanied me. We took her out of bed, and placing her in a broad tub upon a stool, without any other covering than what decency required, poured two pails of cold water over her, after which she was wiped dry, and immediately put to bed. We gave her some wine, and eighty drops of tinct. opii, with orders to repeat the opium, calomel, and wine as before. As soon as she recovered from the alarm, which the first application of this remedy produced, she expressed herself easier, and wished for a repetition of it. At eight in the evening we saw her again; her pulse was then weak and quick; the spasms, though relieved for a time after after the cold affusion, had returned with greater violence; her countenance was dejected, and I thought her worse than ever I had seen her: but, as one application of the cold water could not be thought a fair trial, we agreed to repeat it, and continue the calomel with an additional quantity of opium.

March 7. She passed a more quiet night; and after the cold affusion she was able to stand, and move her limbs, and said she wished she could live in the river, so much better did she feel after its use. She had four motions in the course of the night and this morning; pulse 120. At eight A. M. I saw her again; she was then sick, and vomited her wine and broth immediately after taking them. Cold water and medicines repeated, and strict orders left to repeat the cold affusion at five in the morning.

March 8. At ten this morning I found the sickness had subsided last night soon after the cold affusion; she had had one motion, and was better till about five, at which time the repetition of the water, through the prejudices and obstinacy of the nurses, was neglected. In consequence of this neglect she was in every respect worse, and indeed so considered herself, as she said, I wish my father would let it be done oftener; if he do not, I shall never get well; but the room being very small, and her friends all obstinately against the remedy, it would not, I am persuaded, have been applied half so often, had not Mr. W. C. or myself constantly attended at the hour appointed for its repetition. It was not therefore applied to day till between ten and eleven, again at three in the afternoon, and at nine this evening, when, upon finding her gums were considerably affected by the calomel, this medicine was discontinued, and the dose of opium gradually increased.

March 9. The water was properly applied three times to day. I visited her an hour after the third application and thought her better, the pulse not being more than 100 nor the skin so hot, spasms not so frequent or violent, and the rigidity of the limbs not so great. She had taken plentifully of wine, arrow root, and meat broth, but had had no evacuation by stool since yesterday morning.

March 10. Cold affusion applied three times. She expressed as usual, great relief after each; had slept better last night than she had done since the commencement of the disease, and had one motion in the course of the day. The pulse in the evening were strong, full, and quick, attended with considerable thirst and heat upon the skin. These symptoms of increased fever subsided immediately after after the cold affusion; I ordered her two grains of opium, every three hours, with a draught of decoction of yellow bark strongly impregnated with valerian and camphor.

March 11. Appears to be gradually recovering; has taken plentifully of wine, &c. Cold affusion and medicines as yesterday.

March 12. Continues much the same; has had one evacuation by stool; medicines repeated.

March 13. Not so well; less power over the mouth, and greater rigidity of the back. The effect of the mercurial medicine having disappeared upon the mouth for the last two days, we thought this probably might be the cause of the present unfavourable appearances, and therefore ordered ung. hydrarg. fort. ʒij. to be rubbed in every night, and pil. hydrarg. gr. x. three times a day, with the opium and decoction as before.

March 14. Much as yesterday.

March 15. No amendment; continues to take freely of wine and other nourishment; pulse 104. Ordered to continue the cold affusion and take one of the following pills every three hours: ℞. Pil. hydarg. ʒj. extr. opii gr. xxiv. cons. cynosb. q. s. f. p. No. xij.

March 16. Much as yesterday; continues taking pills and mixture.

March 17. Rested better last night, pulse 100; in other respects the same.

March 18. Had a good night; symptoms of ptyalism are coming on; medicines repeated.

March 19. This morning she did not complain so much of the rigidity of her back, and has more power over her mouth. As the mercurial action appeared to be increasing fast, I ordered gr. ij. of opium to be taken every three hours, alternately with and without the mercury.

March 20. Slept very well last night, and was able this morning, with the support of two people, to walk from the bath to her bed after the cold affusion; having had three loose motions, I ordered a starch enema; mixture and pills continued.

March 21 and 22. Better; bowels regular, sleeps well; the dose of opium and mercury lessened upon repetition.

March 23. She complained much of a sensation of sickness, with disinclination to take nourishment, and she obstinately refused her medicines; her tetanic symptoms, notwithstanding, evidently decrease.

March 24. Last night the sickness was attended with frequent retching, and she took little or no nourishment.

March

March 25. The sickness still continued; and as we could not prevail upon her to take any medicine, an enema, with 120 drops of tinct. opii, was ordered to be thrown up in the evening.

March 26. Rested very well last night, and has consented to take a table spoonful of the following mixture every four hours: ℞. Kali ppt. ʒij. tinct. columb. ℥j. tinct. cardum. ʒij. aq. puræ ℥iij. M. f. misturæ.

March 27. The tetanic symptoms daily diminish, and were it not for the irritability of the stomach, her speedy recovery might I think be reasonably expected. The cold affusion ordered to be used in future in the morning only.

March 28 and 29. The sickness not so troublesome; ordered a table spoonful of the following every four hours: ℞. Magnes. vitriol. ʒiij. tinct. cardum. ʒij. aq. ℥iv. M. f. mist.; all other medicines discontinued.

March 30 and 31. Continues to mend daily; appetite returned, and bowels regular.

April 5. Although very weak, my patient may be considered convalescent.

I cannot conclude this case without thus publicly acknowledging my obligations to Mr. W. H. Crowfoot for the original suggestion of this mode of treatment, and the friendly assistance which he has given me during the application of it.

